# Modeling clock-related metabolic syndrome due to conflicting light and food cues

**DOI:** 10.1038/s41598-018-31804-9

**Published:** 2018-09-11

**Authors:** Aurore Woller, Didier Gonze

**Affiliations:** Unité de Chronobiologie Théorique, Faculté des Sciences, Université Libre de Bruxelles, Campus Plaine, CP 231, B-1050 Brussels, Belgium

## Abstract

Most organisms possess a light- and food- entrainable circadian clock system enabling their adaptation to daily environmental changes in sunlight and food availability. The mammalian circadian system is composed of multiple clocks throughout the body. These local clocks are entrained by nutrient, neural, endocrine and temperature cues and drive diverse physiological functions including metabolism. In particular, the clock of the pancreatic *β* cell rhythmically regulates the transcription of genes involved in glucose-stimulated insulin secretion. Perturbations of this fine-tuned oscillatory network increase the susceptibility to diseases. Besides chronic jet lag and shift work, common perturbations are ill-timed eating patterns which can lead to metabolic troubles (such as hypoinsulinemia). We have built a mathematical model describing the clock-dependent pancreatic regulation of glucose homeostasis in rodents. After calibrating the model using experimental data, we have investigated the effect of restricting food access to the normal rest phase. Our simulations show that the conflict between the light-dark cycle and the feeding-fasting cycle creates a differential phase shift in the expression of core clock genes (consistent with experimental observations). Our model further predicts that this induces a non-concomitance between nutrient cues and clock-controlled cues driving metabolic outputs which results in hypoinsulinemia, hyperglycemia as well as in a loss of food anticipation.

## Introduction

Most living organisms adapt to predictable daily changes in food availability and sunlight thanks to a light- and food-entrainable circadian clock system. These circadian rhythms are cell-autonomous, being generated by transcriptional- translational feedback loops^1^. In mammals, the central components of the cellular oscillator are the activator complex CLOCK-BMAL1 and the repressors PER1-3/CRY1-2. CLOCK-BMAL1 activates the transcription of clock genes, including Per1,2,3 and Cry1,2. PER and CRY proteins associate to form a complex PER-CRY which inhibits the action of CLOCK-BMAL1. CLOCK-BMAL1 also activates the expression of the nuclear receptors REV-ERB*α*, *β* and ROR*α*, *β*, *γ* which respectively inhibit and activate the transcription of *Bmal*1^2^. Circadian oscillators have been identified in multiple tissues including the brain, liver, heart, pancreas and muscles^3^. In mammals, the circadian system is composed of a central clock in the suprachiasmatic nucleus (SCN) of the brain. This pacemaker, which is composed of about 20,000 interconnected neurons each containing the molecular clock machinery, is entrained by the light-dark cycle^4^. In turn, it entrains secondary clocks located in peripheral tissues through neural, endocrine and temperature cues^5^. These peripheral clocks also strongly respond to the alternation between feeding and fasting phases^6^. Local clocks regulate many physiological processes including metabolism^7–10^. More specifically, the clock partitions internal metabolic processes (such as insulin secretion, glucose and lipid storage) to the appropriate phase with respect to external environmental variations^11^. In particular, insulin secretion is not only controlled by nutrient cues but also regulated by the pancreatic *β* cell clock^12,13^. After a meal ingestion, *β* cells uptake and metabolise glucose which leads to an influx of calcium into these cells. This triggers the fusion of insulin-containing granules with the plasma membrane which leads to insulin secretion (also called insulin exocytosis)^14^. It has recently been shown that the transcription of several factors involved in exocytosis is under the control of the *β* cell clock^13^.

**Figure 1:**
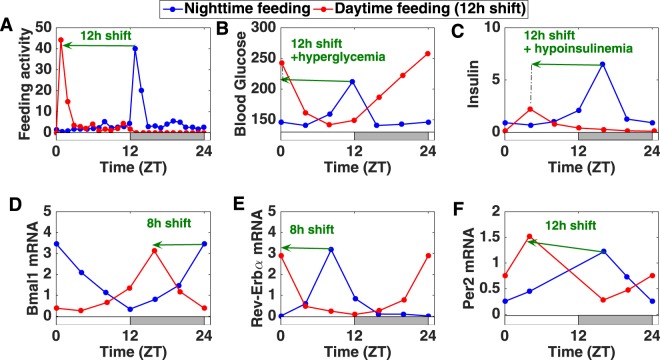
Effect of restricting food access to the normal rest phase in published experimental data (pancreas, mouse)^18,19^. (**A**) Measurement of the feeding activity which corresponds to the number of pellets consumed per hour^19^. (**B**,**C**) Levels of blood glucose and insulin^18^. (**D**–**F**) Pancreatic clock gene expression^18^. Comparison between the normal feeding (in blue) and the time-restricted feeding (in red) conditions. The alternation of white and grey bars symbolises the LD cycles.

The circadian system thus forms a fine-tuned oscillatory network throughout the body. Perturbations such as chronic jet lag or shift work disturb the coordination of this system which increases its susceptibility to diseases^15–17^. Another important perturbation is eating at the wrong time (ill-timed eating patterns). Earlier studies in rodents have shown that shifting the eating schedule by 12 h inverts clock gene expression in the liver and other peripheral tissues while keeping the rhythm unchanged in the central clock^6^. These results led the authors to suggest that when food and light cues are conflicting, local clocks uncouple from the central clock and primarily follow food cues. In recent studies, Mukherji *et al*. have investigated the metabolic effect of shifting mice from nighttime to daytime feeding (Fig. 1A)^18,19^. In this case, the phase of metabolic markers (levels of blood glucose, insulin, free fatty acids,…) and clock gene expression is inverted. Moreover, animals also develop metabolic symptoms such as hypoinsulinemia, hypertriglyceridemia and hyperglycemia (Fig. 1B,C). This leads to the following question: If everything is simply inverted compared to the physiological situation, why do animals exhibit metabolic symptoms? In other words, if the inversion of food access leads to a 12 h phase shift in food intake and peripheral clock gene expression, why is it that nutrient- and clock-responsive gene transcription and insulin secretion is disturbed rather than just shifted by 12 h? A careful observation of experimental data^18^ indicates that while food intake and nutrient cues are shifted by 12 h, there is a gene-specific phase shift in clock gene expression: some genes are shifted by 12 h while others only shift by 8 h (Fig. 1D–F). This 8 h shift in clock gene expression is observed in several tissues including liver and pancreas. These observations raise several questions: what is the mechanism behind this gene-specific phase shift and can the latter explain the metabolic syndrome?

Mathematical modelling can be very useful to understand such puzzling experimental results because it allows a global approach of dynamical systems. So far, only a few modelling studies have focused on the coupling between circadian and metabolic pathways and their interplay with the feeding-fasting cycle and most of them were developped for the liver^20–22^. Among them, Bae *et al*. have explored the effect of circadian disruption of hepatic glucose metabolism^21^ while Woller *et al*. have investigated the effect of metabolic perturbations such as high fat diet feeding on hepatic clock gene expression^20^.

In this study, we focus on the crosstalk between clock and metabolism in the murine pancreatic *β* cell and use mathematical modelling to propose an explanation for the phenotype observed when shifting the eating schedule by 12 h. After calibrating our mathematical model to fit the wild type phenotype (first section), we use it to investigate what happens when food and light cues are conflicting. We hypothesise that peripheral clocks do not completely uncouple from the central when food intake is inverted, contrary to what was earlier assumed^6^ and show that this hypothesis is sufficient to explain the differential phase shift in clock gene expression (second section). In the next sections, we propose a mechanism showing how this differential phase shift leads to disturbed *β* cell metabolism. Our model predicts that this induces a non-concomitance between nutrient cues and clock-controlled exocytosis cues controlling insulin secretion which results in hypoinsulinemia, hyperglyceridemia (third section) as well as in a loss of food anticipation (fourth section). Finally, our model enables us to discuss the role of the widespread circadian control of metabolism by comparing the properties of the physiological network with those of alternative architectures (last section).

## Results

### Construction of a model reproducing the wild-type phenotype

**Figure 2:**
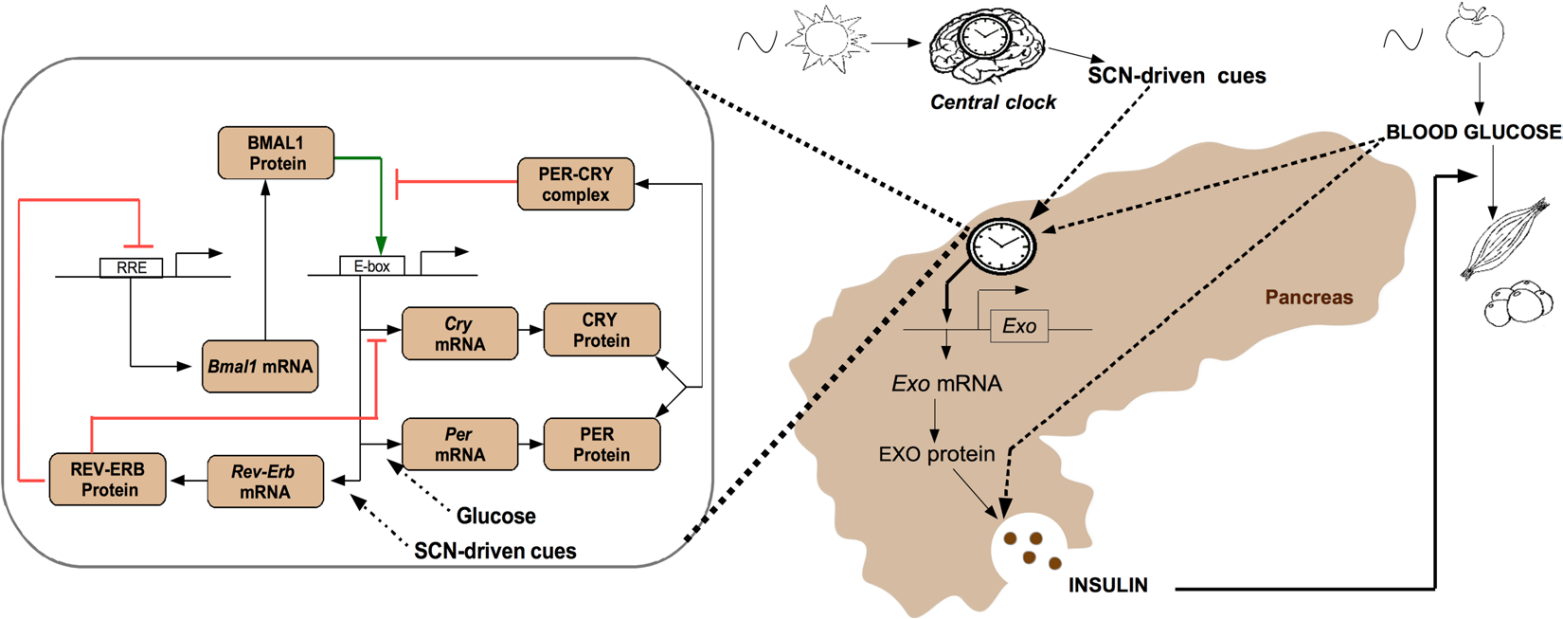
Scheme of the model: Insulin secretion requires the presence of both glucose and the proteins involved in its exocytosis (here represented by EXO protein). The expression of the latter is controlled by the *β* cell clock (mainly via BMAL1). This local clock comprises transcriptional-translational feedback loops shown in the left box and is entrained by both SCN-driven cues and nutrient cues (here represented by glucose).

Our model consists in two embedded networks: The metabolic network composed of the glucose-insulin module and the *β* cell circadian oscillator (Fig. 2). On one side, we model the metabolic module as follows: a 24 h forcing function representing food intake periodically increases blood glucose levels. This stimulates insulin secretion which in turn, lowers blood glucose levels by increasing its uptake by muscles and adipose tissue. On the other side, our clock network model comprises the main transcriptional translational feedback loop with the positive arm (represented by the protein BMAL1) activating the negative arm (represented by *Per* and *Cry)* which in turn shuts down the positive regulation of *Bmal1*. We also model the additional feedback loop between *Bmal1* and *Rev-Erb* because several studies have highlighted the importance of this regulation in the observed phases of clock gene expression^23,24^. To keep the model as simple as possible, we merge the different isoforms of the clock components into one variable and we make no distinction between cytoplasmic and nuclear proteins.

We then add the bi-directional coupling between the clock and the metabolic module. With regard to the circadian control of metabolism, Perelis *et al*. have shown that many transcripts of factors involved in insulin exocytosis are cycling and affected by a knockout of *Bmal1*^13^. This includes factors involved in diverse aspects of insulin exocytosis such as vesicule budding, transport, tethering and fusion. In particular, the authors also showed that BMAL1 and CLOCK regulate the expression of these genes by binding at distal regulatory elements. Therefore, in our model, the coupling of the *β* cell clock to metabolism is mainly achieved via BMAL1. More specifically, for the sake of simplicity, we only model the dynamics of one generic clock-controlled peptide involved in insulin secretion (termed as *Exo* mRNA and EXO protein). Furthermore, we consider that under normal physiological conditions, glucose levels and exocytosis peptide concentration are high simultaneously in order to ensure optimal insulin secretion. In other words, we consider that high insulin secretion is only possible if glucose levels and exocytosis peptide concentration are high simultaneously.

Concerning the control of the clock by metabolism, very little is known about how nutrient cues affect the *β* cell clock. Since increasing glucose concentration increases *Per* transcription in cultured rats islets^25^, we here assume that glucose cues act on *Per* transcription. As we will see in the next section, the 12 h shift of *Per* mRNA when restricting food access to the normal rest phase can thus be explained by the 12 h shift of glucose cues.

Finally, as we aim to use the model to study what happens when light and food cues are conflicting, we must incorporate the effect of these cues in our system. Information about the feeding cues is transmitted to the system through glucose levels that rise after a meal. Light information is transmitted from the centre to the periphery through SCN-driven outputs which include endocrine, temperature and neural cues. Under daytime feeding, the time profiles of endocrine cues such as glucocorticoids (as well as those of body temperature) become bimodal, that is, one peak is shifted by 12 h and the other peak is not shifted)^6,18,26^. This is probably due to the fact that these cues receive information both from the non-shifting SCN and from the shifting nutrient cues. Although glucocorticoid signalling is known to act on *Per* transcription^27^, the bimodal profile does not seem to affect much *Per* time profile^26^. Therefore and also because the effect of nutrient cues on *Per* are sufficient to explain its 12 shift, for the sake of simplicity, we do not incorporate glucocorticoid signalling in the model. Finally, very little is known about the time profile of neural cues after an inversion of the feeding schedule. To explain the partial phase shift of the other clock genes, we hypothesise that there is at least some SCN-driven signal that is not shifted when inverting the eating schedule: this could be the case of neural cues. Since the rhythm of neuronal activity peaks during the light period^28^, we consider that it increases *Rev-Erb* expression because the latter is raising during the subjective day.

**Figure 3:**
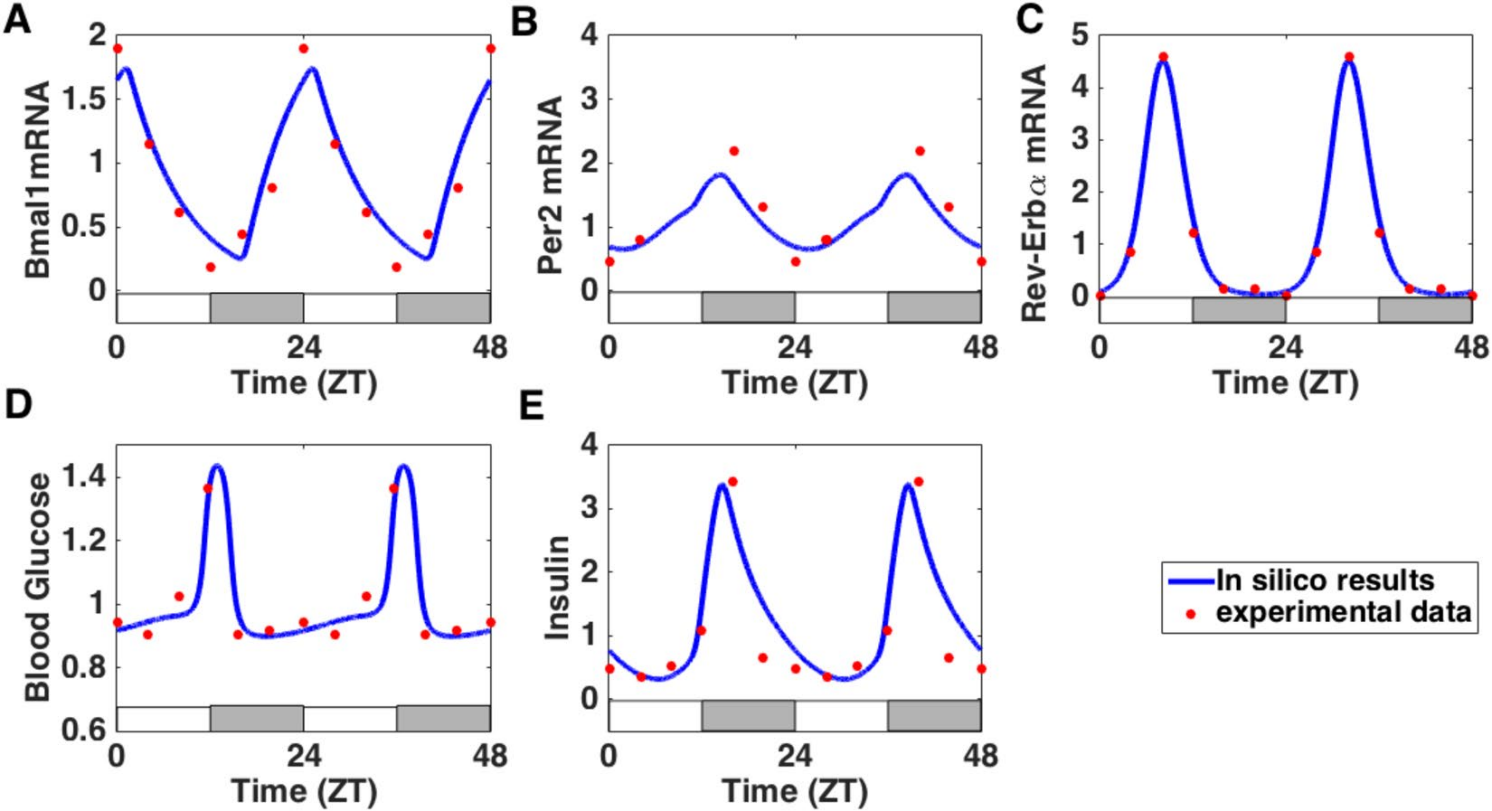
The model reproduces the wild-type phenotype. (**A**–**E**) Comparison of *in silico* time series (blue solid line) with experimental time profiles^18^ (red dots, replicated to cover two days). The alternation of white and grey bars symbolises the LD cycles. The equations of the model are given in the Supplementary Information and the fitting procedure is described in the Method section.

Figure 2 summarises the interaction network considered in our model. The model consists in 13 ordinary differential equations governing the dynamics of the key variables of our system (9 clock components, glucose, insulin and EXO mRNA/protein), as well as two periodic signals, standing for the SCN and the food cues (see Supplementary Information). In order to properly reproduce experimental time profiles of pancreatic clock gene expression and metabolic factors levels, we fit our mathematical model to published experimental data coming from mice kept under 12 h light conditions and fed *ad libitum*^18^. Figure 3 shows that the *in silico* time profiles correctly match the experimental ones for both metabolic and *β* cell clock components.

### Differential phase shift in clock gene expression due to conflict between nutrient and SCN-driven cues

**Figure 4:**
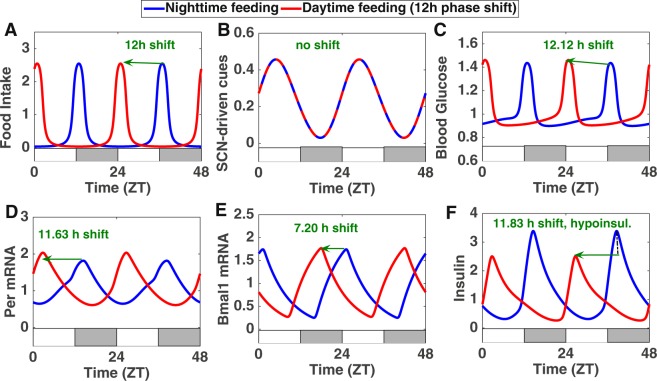
Effect of a shift from nighttime feeding (NF, blue curves) to daytime feeding (DF, red curves): when food intake (shifted by 12 h, panel A) and SCN-driven cues (not shifted, panel B) are conflicting, some components are shifted by almost 12 h (panels C,D and F) while others are shifted much less (panel E). This also leads to hypoinsulinemia (panel F). The alternation of white and grey bars symbolises the LD cycles.

We then investigate the effect of restricting food access to the light period. We simulate this perturbation by shifting the phase of the forcing function representing food intake by 12 h while leaving the one representing SCN-driven cues unchanged (Fig. 4A,B). Our hypothesis is that the resulting conflict between nutrient and SCN-driven inputs driving the local clock could explain the observed differential phase shift in clock gene expression. We were able to find a set of parameter values that qualitatively reproduce experimental data: *Per* expression shifts by 11.63 h while the phase shift of the other clock genes (*Bmal1*, *Rev-Erb* and *Cry* mRNAs) lies between 7 and 9 h (Figs 4 and S1). This differential phase shift suggests that the phase relationship between the positive and the negative arm of the clock is not preserved when several systemic inputs are conflicting.

**Figure 5:**
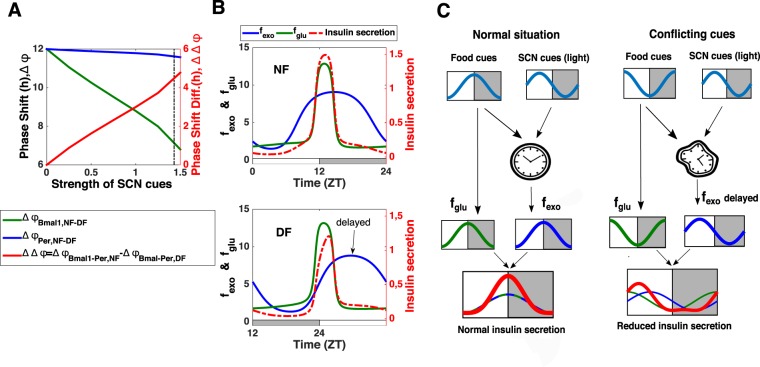
Mechanism behind the metabolic syndrome: differential phase shift in local clock gene expression due to conflict between nutrient and SCN-driven cues and resulting misalignment between nutrient and clock-controlled cues. (**A**) Left Y-axis: Effect of a shift from NF to DF as a function of the strength of SCN-driven cues for *Per* mRNA (phase shift in blue) and *Bmal1* (phase shift in green). The strength of the SCN-driven cues corresponds to the value of the parameter *cneur* (see Supplementary Information) and it is altered by increasing the parameter value from 0 to 1.55. The vertical black dotted line corresponds to the strength of SCN-driven cues used in the rest of the simulations (*cneur* = 1.4336). Right Y-axis: The phase shift difference between *Bmal1* and *Per* mRNAs (which corresponds to the phase difference *Bmal*-*Per* in NF minus the phase difference *Bmal*-*Per* in DF) is also represented as a function of the strength of SCN-driven cues (red curve). (**B**) Upper panel: Insulin secretion (red dotted curve) for nighttime feeding: the high secretion results from an alignment between glucose cues (*f*_*glu*_, green curve) and clock-controlled exocytosis cues (*f*_*exo*_, blue curve). Lower panel: Insulin secretion (red dotted curve) for daytime feeding: the reduced secretion results from a misaligment between glucose cues (*f*_*glu*_, green curve) and clock-controlled exocytosis cues (*f*_*exo*_, blue curve) which are delayed. The quantities *f*_*exo*_ and *f*_*glu*_ represent the effect on insulin secretion of glucose and clock-controlled exocytosis cues respectively (see Table S2 in Supplementary Information for the exact expressions). The alternation of white and grey bars symbolises the LD cycles. (**C**) Summary of the mechanism leading to hypoinsulinemia when nutrient and SCN-driven cues are conflicting.

To test this more systematically, we progressively increase the strength of SCN-driven cues and compare the phase shift of several clock components when inverting the eating schedule (Fig. 5A). In the absence of SCN-driven cues, the whole clock shifts by 12 h. When increasing the strength of SCN-driven cues, the simulations show that the different clock components do not shift in the same way. Indeed, the negative element *Per*, which receives information about food intake (through Glucose cues), shifts by almost 12 h regardless of the strength of the SCN cue. On the other hand, the positive element *Bmal1*, which also integrates the unchanged SCN-driven cues through *Rev-Erbα*, undergoes smaller phase shifts as the strength of the SCN cues increases. As we will see in the next section, this can impact the phase and amplitude of clock-controlled physiological outputs.

One might wonder if this “differential phase shift” property depends on a specific parameter set. A further analysis shows how the phase shift of *Bmal1* and *Per* is influenced by the variations of a few relevant parameters (see supplementary material, section 3.2 and Fig. S2). This analysis indicates that this “differential phase shift” property does not depend on a specific set of parameter values and suggests that it could be more general for the described model.

Until now, we have considered large perturbations such as a 12 h phase shift in food intake. We then investigate the effect of smaller perturbations in the eating schedule: The simulations show that the phase difference between different clock components is only slightly changed for a short advance or delay in food intake (Fig. S3A,B). Altogether, the equilibrium between the positive and the negative arm can be strongly affected by large chronic perturbations.

### Metabolic syndrome due to misalignment between nutrient and clock-driven cues

In order to ensure a timely insulin secretion, the clock-controlled protein machinery participating in insulin exocytosis has to be present when nutrients (glucose) are ingested. Therefore, we assume that a high insulin exocytosis requires the concomitance between glucose and clock-controlled exocytosis cues. This condition is integrated in the model by describing the time evolution of insulin levels as (see also Eq. S17 in the Suppl. Mat.)

1 $$\frac{d[Insulin]\,}{dt}={k}_{i}\cdot {f}_{glu}\cdot {f}_{exo}-{d}_{i}\cdot [Insulin]$$

where the first term of the right hand side represents insulin secretion and the second term, insulin clearance. The kinetic constant *k*_*i*_ is the maximal secretion rate and *d*_*i*_ is the clearance constant. The function *f*_*exo*_ = *f(clock)* represents the effect of clock-controlled exocytosis cues on insulin secretion and its dynamics is mainly driven by BMAL1 while *f*_*glu*_ = *f(glucose)* corresponds to the effect of glucose on insulin secretion (see Table S2 in Supplementary Information for the exact expressions of these functions (*f*_*Ins,EXO*_ and *f*_*Ins,glucose*_)).

Thus, under normal feeding conditions, the peak in the concentration of factors involved in insulin exocytosis is concomitant with the peak in blood glucose levels, thereby ensuring optimal insulin secretion (Fig. 5B, upper panel). However, disturbing the phase relation between the different core clock genes can strongly affect the phase of clock-controlled outputs. Indeed, our simulations show that the partial shift of some clock components following a reversal of the eating schedule also results in an incomplete inversion of the clock-controlled expression of exocytosis peptides. Consequently, the rise in the concentration of the factors involved in insulin exocytosis is now delayed and no longer concomitant with the rise in blood glucose levels (Fig. 5B, lower panel). This results in a reduced insulin secretion and consequent increase in blood glucose levels (Fig. 4C,F). Thus our model shows that the metabolic syndrome (hypoinsulinemia and hyperglycemia) observed during restricted feeding can be explained by a disruption of the coincidence mechanism between clock-controlled and nutrient cues due to upstream conflicts between SCN-driven and nutrient cues (Fig. 5C). Our simulations also predict that there is no significative amplitude change in insulin secretion for smaller shifts in the eating schedule (Fig. S3C,D).

We further use the model to test what happens when peripheral clocks completely uncouple from the central when food intake is inverted, as it was earlier assumed^6^. The model predicts that in this case, all genes would shift by 12 h when inverting the eating schedule and that insulin levels would not be reduced, which is not what is observed experimentally (Fig. S4). Thus, the hypothesis of a complete uncoupling from the SCN fails to reproduce the differential phase shift in clock gene expression as well as the metabolic syndrome.

### Loss of anticipation due to misalignment between nutrient and clock-driven cues

**Figure 6:**
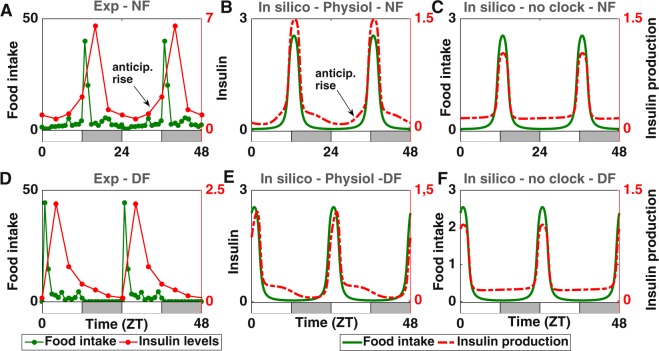
Loss of anticipation due to misalignment between nutrient and clock-controlled exocytosis cues. (**A**,**D**) Comparison of experimental insulin levels (red dots) for nighttime (upper panel, A) and daytime feeding (lower panel, D). (**B**,**E**) Comparison of *in silico* insulin secretion (red dotted curve, B) for nighttime (upper panel, E) and daytime feeding (lower panel). (**C**,**F**) Comparison of *in silico* insulin levels (red dotted curve) for nighttime (upper panel, C) and daytime feeding (lower panel, F) in the absence of any clock. Food intake is represented in green. A food anticipatory insulin rise can only be observed in panels A and B. The alternation of white and grey bars symbolises the LD cycles.

It is commonly assumed that the cross-talk between clock and metabolic pathways enables the anticipation of the “daily” (periodic) arrival of food. This raises the question of whether this property can be observed in clock-controlled metabolic outputs and how it is affected upon ill-timed feeding. Under normal (nighttime) feeding conditions, we notice that experimental profiles of insulin levels display a slope rupture (Fig. 6A). Indeed, prior to food intake, the slope of the insulin profile rises slowly, while it increases steeply after meal ingestion. The same feature is reproduced in our simulations (Fig. 6B). Interestingly, this slope rupture is lost in both *in silico* and in experimental data when restricting food access to the light period (Fig. 6D,E). This can be interpreted as follows: in physiological conditions, the clock induces the expression of exocytosis peptides in anticipation of the forthcoming arrival of food, leading to a slow increase of insulin secretion. This is followed by a fast increase in insulin secretion after food intake. When inverting the eating schedule, the clock control of exocytosis is delayed and therefore fails to induce peptides synthesis prior to food intake: this situation corresponds to a passive response to meal ingestion (no anticipatory rise) similar to what would happen in the absence of any clock (see next section). This suggests that the slope rupture observed in physiological metabolic time profiles can be seen as the hallmark of a food anticipatory behaviour due to the circadian control of metabolism.

### Tradeoff between anticipation and adaptation

**Figure 7:**
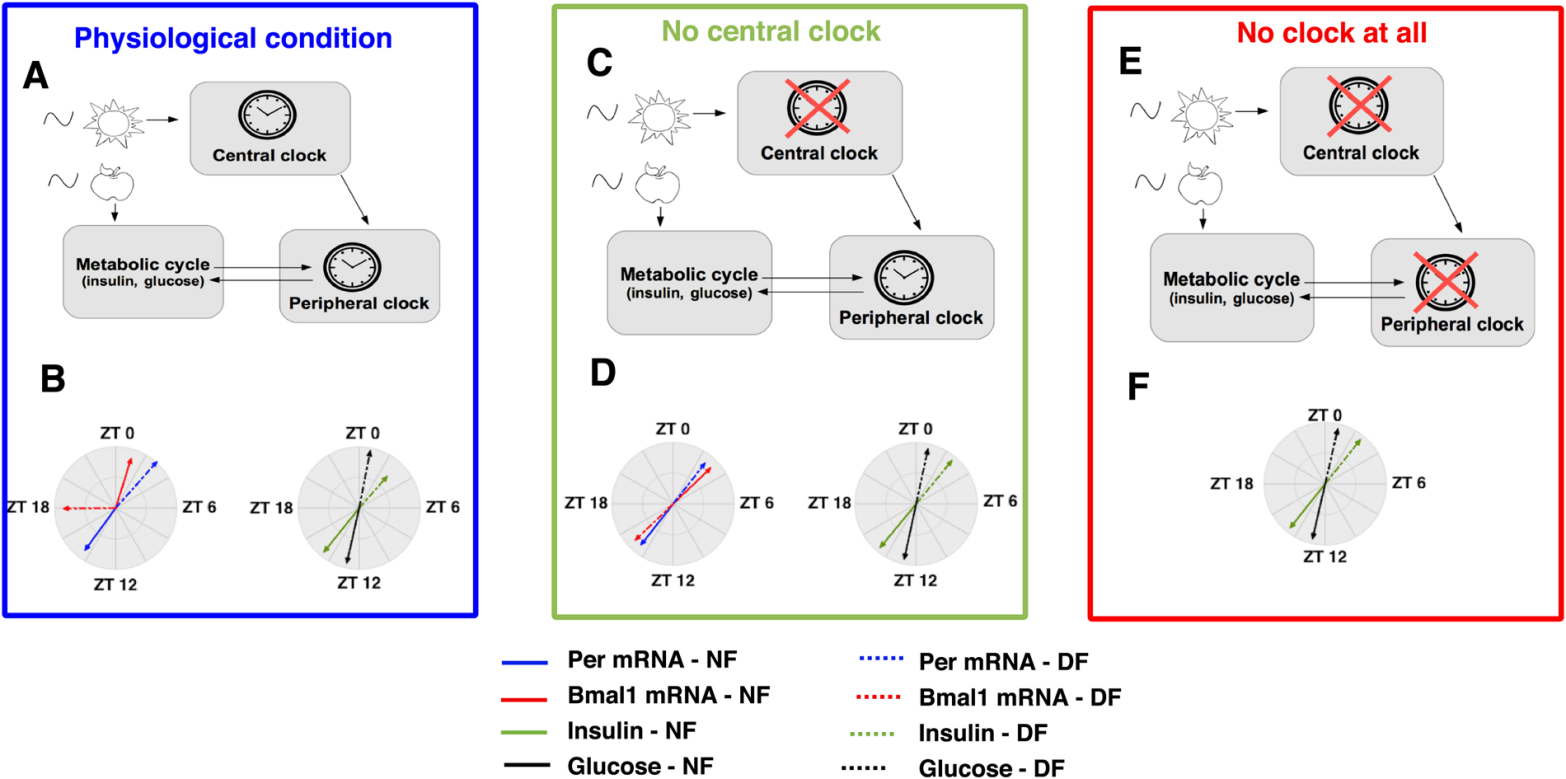
Effect of an inversion in the eating schedule for different architectures. The circular plot summarises the effect of a shift from nighttime feeding (NF) to daytime feeding (DF) on the phase (arrow direction) and on the amplitude (arrow length) of clock genes and metabolic variables in 3 conditions. (**A**,**B**) In the “Physiological” condition (where the local clock entrained by the central pacemaker controls the expression of exocytosis factors, panel A), there is differential phase shift in clock gene expression (compare dotted and solid red arrows) leading to hypoinsulinemia (reflected here by the reduced length of the dotted green arrow). (**C**,**D**) In the “No central clock” condition (where the local clock controls the expression of exocytosis factors, panel C), all genes shift by 12 h and the level of insulin is the same during nighttime and daytime feeding (panels D). (**E**,**F**) In the “No clock at all” condition (where the expression of exocytosis factors is not under the control of the clock, panel E), the levels of the metabolic variables are the same during nighttime and daytime feeding (panel F).

Finally, our mathematical model allows us to discuss the implications of the circadian control of metabolism. A way to gain insight into the properties of a particular physiological network is to compare it to alternative architectures, a task for which mathematical modelling is particularly well-suited. In this regard, here we compare the physiological architecture described above (a local clock driven by SCN cues and coupled to a metabolic cycle) to two alternatives: the case where the metabolic module is only controlled by the local clock (that is, without any rhythmic cues from the SCN informing about the light-dark cycle) and the case where the metabolic module is not controlled by any clock. We refer to the first situation as “physiological case” (Fig. 7A), the second one as “no central clock” (Fig. 7C) and the last one as “no clock at all” (Fig. 7E). The absence of a central clock is simulated by replacing the SCN-driven rhythm by its mean value. Similarly, we mimic the absence of a local clock by replacing the circadian transcription of the factor involved in exocytosis (here *Exo* mRNA) by its mean value (see Supplementary Information).

Having a clock system that is sensitive to light cues and nutrient cues (physiological and “no central clock” cases) enables the anticipation of predictable environmental changes. By having a local clock that is sensitive to nutrient cues and controls metabolic outputs, organisms are able to anticipate food availability and metabolise the ingested nutrients optimally (Fig. 6A,B). In the absence of any clock, they can only passively respond to food intake (no anticipation): this is characterised by an absence of food anticipatory rise in metabolic time profiles (Fig. 6C). In addition, having a light anticipatory mechanism as in the physiological case enables the organism to keep track of the alternation between active and rest phases so that starving animals can still look for food during the appropriate phase of the cycle^18^.

However, this anticipation property comes with a cost: as we have seen in the previous sections, in physiological conditions, the organism is not able to completely adapt to large chronic perturbations in food intake, which creates metabolic syndrome-like pathology. Figure 7B summarises this prediction with a circular plot representation: an inverted eating schedule leads to a differential phase shift in clock gene expression and to subsequent hypoinsulinemia. Conversely, in the absence of light information from the SCN (“no central clock” architecture), the system would completely adapt to the new feeding schedule because the local clock would not be twisted by conflicting light and food cues. This is illustrated in Fig. 7D (no differential phase shift of clock components and similar insulin levels during nighttime and daytime feeding). But the adaptation of such a system is predicted to take some time because of the inertia of the local clock (Fig. S5A,B and E). In contrast, in the absence of any clock (“no clock at all” situation), the metabolic response to large chronic changes in food availability is immediate (Fig. S5C–E). Altogether, these observations suggest that there is a tradeoff between the ability to sense light and nutrient cues (which leads to anticipation) and the ability to fully adapt to chronic perturbations in one of these inputs.

## Discussion

In the 24/7 culture of the modern world, our organism is subjected to diverse perturbations (including chronic jet-lag, shift work and ill-timed eating patterns) which affect the pace of our circadian system and can lead to pathologies. The complete understanding of this dynamical system requires a holistic approach for which mathematical modeling can be very beneficial. Here, we have built a mathematical model describing the clock-dependent pancreatic regulation of glucose homeostasis in rodents. After calibrating the model, we have investigated the effect of restricting food access to the normal rest phase. Our simulations have shown that the resulting conflict between the light-dark cycle (represented here by SCN-driven cues) and the feeding-fasting cycle (represented here by nutrient (glucose) cues) creates a kind of distortion of the core clock, with some clock components following the eating schedule (completely inverted expression) and others being twisted between the conflicting inputs (only partially inverted expression). This can thus explain the differential phase shift in the expression of core clock genes that we have noticed in published data^18^. We have shown that these phase alterations in the local clock can cause a downstream domino effect which has several consequences: the loss of anticipatory “behaviour” and an amplitude reduction of insulin due to the disruption of the coincidence mechanism between nutrient and clock-controlled exocytosis cues. The consequences of this hypoinsulinemia are important: it leads to increased blood glucose levels and decreased fat storage which can in turn affect other fundamental metabolic processes. Our work thus highlights that the desynchronisation between light and food cues has a cascading effect which can affect the whole metabolism and increases animals susceptibility to diseases.

We believe that the mechanism that we have underlined is more general than the pancreatic case we have studied here. Indeed, other peripheral metabolic tissues comprise a local clock which receives diverse systemic cues^29,30^ that can potentially conflict: in the same work of Mukherji *et al*., the other studied tissues (such as the liver, the intestinal epithelial cells or the paraventricular nucleus but not the SCN) also present a differential phase shift in their core clock components when restricting food access to light phase^18^. Furthermore, similar results can be found in other recent studies^31,32^: for example, Takahashi and coworkers have recently shown that the skin clock and skin clock-controlled genes shift only partially or not at all when inverting the eating schedule^32^. In older studies, cycling hepatic transcripts also show differential phase shift when animals are switched from *ad libitum* to daytime restricted feeding conditions^33^. One could argue that this phenomenon was not observed in pioneer studies about day time-restricted feeding in rodents but this is probably due to the very low time resolution in clock gene expression profiles.

So far, only a few computational models have described the coupling between the mammalian circadian clock and metabolism, mainly for the liver^20–22^. Our model provides a framework for a better understanding of the dramatic metabolic consequences of a desynchronisation between light and food Zeitgebers. However, the proposed mechanism is unlikely to entirely explain the metabolic syndrome observed when restricting food intake to the normal rest phase. Indeed, insulin secretion also strongly relies on *β* cell function (islet size and proliferation) which itself appears to be clock-controlled^12^. Furthermore, insulin also feeds back from the periphery to the centre to modulate food intake^34^. Another important point is that glucose levels are also regulated by the action of glucagon whose secretion is controlled by the *α* cell clock^35^. Therefore, it would be interesting to incorporate these additional levels of complexity in further work. More generally, it would be worth to couple the model to circuits controlling cell growth, proliferation, damage responses and cell death to investigate how the clock influences cell fate decisions.

To conclude, by comparing the physiological system to alternative architectures, our work reinforces the idea that the reciprocal coupling between clock and metabolism probably constitutes an evolutionary strategy ensuring the proper coordination of internal metabolic processes with external periodic events. But at the same time, this tight coupling also causes a vulnerability to diseases because the system cannot handle chronic ill-timed behaviours.

## Methods

### Model fitting and simulations

The system of 13 ordinary differential equations (+2 ordinary differential equations for food intake) was implemented in MATLAB R2015a (MathWorks, https://nl.mathworks.com) and solved with ode15s. In order to reproduce expression and metabolic time profiles, the values of the parameters of the model were fitted by the Hooke & Jeeves pattern search optimisation method using COPASI 4.20^36^. The published time profiles come from mice kept under 12 h light conditions and fed *ad libitum* (for the restricted feeding conditions, mice could access food only during the light phase)^18^. These raw data were interpolated with Fourier series in order to facilitate the adjustment with *in silico* time profiles. More detailed information can be found in Supplementary Information.

## Electronic supplementary material

Supplementary Information
