# Supplementary material for: Modeling clock-related metabolic syndrome due to conflicting light and food cues
Source: Sci Rep. 2018 Sep 11;8:13641. doi: 10.1038/s41598-018-31804-9 (PMC6134130; doi:10.1038/s41598-018-31804-9)
Supplement: Supplementary file 1 — Supplementary Information [file 41598_2018_31804_MOESM1_ESM.pdf]

Modeling clock-related metabolic syndrome due to conflicting light and food cues

Supplementary Information

Aurore Woller and Didier Gonze

Unité de Chronobiologie Théorique, Faculté des Sciences, CP 231, Université Libre de Bruxelles, Bvd du Triomphe, B-1050 Brussels, Belgium.

August 20, 2018

Contents

1 Model building **2**

1.1 Equations for the core circadian clock 2

1.2 Equations for the systemic cues driving the clock 3

1.2.1 Non-shifting SCN cues 4

1.2.2 Nutrient cues 4

1.3 Equations for the clock-controlled exocytosis 5

1.4 Equations for Insulin and Glucose 6

2 Derivation of transcription functions **8**

3 Parameter values **10**

3.1 Parameter estimation 10

3.2 Sensitivity to parameter values 10

4 Supplementary figures **14**

1 Model building

Our purpose was to obtain a mathematical model as simple as possible, but able to reproduce experimental phenotypes. To this end, we have built a model composed of 13 ordinary differential equations describing the time evolution of the concentration of clock components as well as of elements of the glucose-insulin module. The variables of the model are defined in Table S1. A few additional equations are used to form the external forcing functions representing food intake (meal) and SCN-driven cues.

	Variable	Description
Clock	$[Per]$	Concentration of *Per* mRNA
	$[Cry]$	Concentration of *Cry* mRNA
	$[Rev]$	Concentration of *Rev-Erb* mRNA
	$[Bmal]$	Concentration of *Bmal* mRNA
	$[PER]$	Concentration of PER protein
	$[CRY]$	Concentration of CRY protein
	$[REV]$	Concentration of REV-ERB protein
	$[BMAL]$	Concentration of BMAL1 protein
	$[PC]$	Concentration of PER-CRY protein complex
Metabolism	$[Insulin]$	Concentration of Insulin
	$[Glucose]$	Concentration of Glucose
	$[Exo]$	Concentration of exocytosis factors mRNA
	$[EXO]$	Concentration of exocytosis factors protein

Table S1: Definition of the variables of the mathematical model.

1.1 Equations for the core circadian clock

Our minimal core clock model comprises two interlocked transcriptional-translational feedback loops (see Figure 2 in the main text). More specifically, the activator, BMAL1 induces the transcription of *Per*, *Cry*, and *Rev-Erb* (Eqs. S1, S3 and S8 respectively) [2]. Their respective mRNAs are translated into proteins (Eqs. S2, S4, and S9). Proteins PER and CRY associate to form a complex, PER-CRY (Eq. S5), which inhibits the action of BMAL1. Protein REV-ERB inhibits the transcription of *Bmal1* (Eq. S6). Furthermore, REV-ERB also inhibits the transcription of *Cry* (Eq. S3) [9]. Our core clock model thus consists of 9 variables (Eqs. S1-S9): 4 mRNAs (*Per*, *Cry*, *Bmal*, and *Rev-Erb* mRNAs), 4 proteins (PER, CRY, BMAL1, and REV-ERB proteins) and 1 complex (PC).

For the sake of simplicity, we have made several approximations. The different isoforms of each clock component were lumped together: *Per*1,2,3 are merged into one variable, *Per*, and *Cry*1,2 are merged into *Cry*. Furthermore, we do not distinguish the cytoplasmic and nuclear forms of each protein. In addition, we neglect some core clock components. First of all, the activator CLOCK was not explicitely modeled because its expression is generally considered to be constitutive [10]. We thus suppose that it is the protein BMAL1 that is driving the dynamics of the positive arm of the clock: this enabled us to reduce the number of equations by considering that BMAL1 is playing the role of the complex

CLOCK-BMAL1. We also do not incorporate the nuclear receptor ROR. Indeed, the latter seems to have a less important role than the nuclear receptor REV-ERB as it does not oscillate in every tissue [19]. Despite its simplicity, the model successfully reproduces experimental time series and phenotypes.

The transcription functions are detailed in Table S2 and reflect the molecular regulation of the core clock genes transcription: the activator complex CLOCK-BMAL1 (here represented by the protein BMAL1) first binds on the promotor of clock-controlled genes. This establishes a transcriptionally active state. Then, the repressors PER and CRY arrive which sets the beginning of a repressive state [7]. The transcription functions (Table S2) based on Hill functions were first successfully used in [14] and then in [18]. In section 2, we show how they can be derived using the quasi-steady state assumption. It should be noted that a basal gene transcription is also allowed which is reflected in the fold change parameters; g_X (that represents the ratio of gene expression (e.g. transcription rate) in the presence and absence of transcription factors [1]).

$$\frac{d[Per]}{dt} = vmP \cdot f_{Per,clock} \cdot f_{Per,nutrient} - dmP \cdot [Per] \quad \text{(S1)}$$

$$\frac{d[PER]}{dt} = kpP \cdot [Per] - dpP \cdot [PER] - kass \cdot [CRY] \cdot [PER] + kdiss \cdot [PC] \quad \text{(S2)}$$

$$\frac{d[Cry]}{dt} = vmC \cdot f_{Cry,clock} - dmC \cdot [Cry] \quad \text{(S3)}$$

$$\frac{d[CRY]}{dt} = kpC \cdot [Cry] - dpC \cdot [CRY] + kdiss \cdot [PC] - kass \cdot [CRY] \cdot [PER] \quad \text{(S4)}$$

$$\frac{d[PC]}{dt} = kass \cdot [CRY] \cdot [PER] - kdiss \cdot [PC] - dgPC \cdot [PC] \quad \text{(S5)}$$

$$\frac{d[Bmal]}{dt} = vmB \cdot f_{Bmal,clock} - dmB \cdot [Bmal] \quad \text{(S6)}$$

$$\frac{d[BMAL]}{dt} = kpB \cdot [Bmal] - dpB \cdot [BMAL] \quad \text{(S7)}$$

$$\frac{d[Rev]}{dt} = vmR \cdot f_{Rev,clock} \cdot f_{Rev,SCN} - dmR \cdot [Rev] \quad \text{(S8)}$$

$$\frac{d[REV]}{dt} = kpR \cdot [Rev] - dpR \cdot [REV] \quad \text{(S9)}$$

1.2 Equations for the systemic cues driving the clock

Peripheral clocks are synchronised to environmental changes by several systemic cues. Among them are nutrient, endocrine, temperature and neural cues which are all under the control of the central clock under normal physiological condition. In our model, these systemic cues act on the transcription of core clock genes (see main text and $f_{Per,nutrient}$ and $f_{Rev,SCN}$ in Eqs. S1 and S8). As the activation of clock gene expression by systemic

cues generally occurs at sites that are distinct from those of CLOCK-BMAL1 regulation (see [16]), the function describing systemic depend-transcription is multipled by the one describing the clock-dependent transcription.

1.2.1 Non-shifting SCN cues

As the maximal neural activity occurs peaks during the light phase [5], we model this SCN output by a Fourier series with the appropriate phase:

$$SCN\_cues = \begin{cases} f_{SCN} & \text{with SCN cues} \\ f_{noSCN} & \text{without SCN} \end{cases} \tag{S10}$$

with $f_{SCN} = a_0 + \sum_{n=1}^{2} a_n \cos\left(\frac{2\pi * n * t}{period}\right) + \sum_{n=1}^{2} b_n \sin\left(\frac{2\pi * n * t}{period}\right)$ and $f_{noSCN} = a_3$.

The parameter values are *period*= 24h, a_0=0.2726, a_1=-0.16, b_1=0.14, a_2=-0.01, b_2=-0.01 and a_3=0.25.

1.2.2 Nutrient cues

In our model, food intake is modelled by a periodic forcing function which qualitatively reproduces the number of pellets that animals consume per hour when food access is *ad libitum*. The feeding activity pattern corresponds to the alternation between starvation phases at the beginning of the light phase and feeding phases at the beginning of the dark phase [11]. To model this specific pattern (see Figure 1A in the main text), we choose a flexible forcing function, *meal*, which can reproduce a wide range of waveforms:

$$meal = cfood \cdot \left(0.5 + \frac{\arctan\left(coef \cdot arg\right)}{\pi}\right) \tag{S11}$$

$$\tag{S12}$$

with

$coef = \frac{steep \cdot period^2}{2 \cdot \pi \cdot dur}$ and $arg = [y] \cdot \cos\left(\frac{2 \cdot \pi \cdot pha}{period}\right) + [x] \cdot \sin\left(\frac{2 \cdot \pi \cdot pha}{period}\right) - \cos\left(\frac{\pi \cdot dur}{period}\right)$.

The variables x and y used in the upper expressions are solutions of the ordinary differential equations:

$$\frac{dx}{dt} = \left(x + y\frac{2\pi}{period}\right) - x\left(x^2 + y^2\right) \tag{S13}$$

$$\frac{dy}{dt} = \left(y - x\frac{2\pi}{period}\right) - y\left(x^2 + y^2\right) \tag{S14}$$

The parameter values are *cfood*=3.29, *period*= 24h, *steep*= 0.5, *dur*=3h and *pha*=17h for nighttime feeding and 5h for daytime feeding.

We use this forcing function because it is very flexible and this allows the comparison between different food-access paradigms such as *ad libitum* food access (*ad lib*) versus food access restricted to the night (tRF). Panda et al have shown that for tRF, the food intake function is more narrow and has a higher amplitude compared to the *ad lib* situation [4]. Our simulations show that the expression of some clock genes as well as insulin levels are boosted for a steeper food intake function and this is in agreement with Panda' results [4] (Figure S6).

1.3 Equations for the clock-controlled exocytosis

It has been shown that BMAL1 and CLOCK bind to enhancers of genes coding for diverses factors involved in insulin exocytosis ([12]). These elements are involved in the budding, transport, tethering and fusion of vesicles participating in insulin secretion. The phases of these cycling transcripts are variable: some of them peak 8h after *Bmal1* mRNA while others peak at the transition between light and dark. The earlier elements include for example *Vps13a,c,d* which affect trafficking [12]. The latter elements include transcripts of factors such as *Rhoa* and *Rhob* that activate the protein kinase C (PKC) which contribute to initiate vesicle fusion [12]. Here, for the sake of simplicity, we only model the dynamics (mRNA and protein) of one generic element involved in exocytosis that we call Exo: we assume that its transcription is driven by the positive element BMAL1 and repressed by the negative arm of the clock (Eq. S15). We use a transcription function ($f_{Exo,clock}$) that is similar to those used for the core clock (see Table S2). The mRNA Exo is then translated into a protein, EXO (Eq. S16). While we did not constrain the parameters describing *Exo* mRNA dynamics, we did for those of EXO protein because we wanted its phase to be such that the EXO protein and glucose cues act simultanously to induce optimal insulin secretion under normal physiological conditions.

$$\frac{d[Exo]}{dt} = \begin{cases} vmE \cdot f_{Exo,clock} - dmE \cdot [Exo] & \text{with clock} \\ vmE0 - dmE \cdot [Exo] & \text{without clock} \end{cases} \tag{S15}$$

$$\frac{d[EXO]}{dt} = kpE \cdot [Exo] - dpE \cdot [EXO] \tag{S16}$$

It should be mentioned that the β cell clock likely regulates insulin exocytosis in multiple ways. While in our model, the coupling of the clock to metabolism is mainly achieved via BMAL1 in agreement recent experimental results [12]), it is known that some exocytosis-related genes are also regulated by REV-ERBα. Indeed, Vieira et al 2012 showed that the expression of some exocytosis-related factors is reduced after a knockdown of *Rev-Erb*α [17] but modeling these specific regulations in more detail is beyond the scope of this study. Instead, our view was to keep our model as simple as possible (one generic clock-controlled exocytosis factor), focusing on the important point that the maximum accumulation of our generic exocytosis factor has to coincide with the peak of glucose.

1.4 Equations for Insulin and Glucose

We model insulin dynamics as the balance between its secretion and its clearance (Eq. S17). Insulin secretion is modelled as a sigmoidal function of glucose levels which is consistent with experimental data (see references in [15]). We also use a sigmoidal function for the effect of the protein EXO on insulin secretion to account for the complexity of the exocytosis process. We consider that both the levels of glucose and exocytosis proteins (EXO) have to be high to promote a high insulin secretion: this corresponds to an AND gate and therefore, we multiply the functions describing the effect of glucose and EXO on insulin secretion by each other (see $f_{Ins,glucose}$ and $f_{Ins,EXO}$ in Table S2).

We model blood glucose dynamics as a balance between its production and its uptake (Eq. S18). Glucose production is modelled as the sum of a basal production rate and a meal-dependent production rate. We consider that the basal production rate incorporates *inter alia* the glucagon-dependent rise of blood glucose. Glucose uptake is written as a michaelian function of insulin level.

$$\frac{d[Insulin]}{dt} = kins \cdot f_{Ins,glucose} \cdot f_{Ins,EXO} - dins \cdot [Insulin] \tag{S17}$$

$$\frac{d[Glucose]}{dt} = kg + meal - \left(dg + \frac{di \cdot ([Insulin])}{Kinsu + [Insulin]} \right) \cdot [Glucose] \tag{S18}$$

Function	Expression
$f_{Per,clock}$	$\frac{1+g_P\cdot\left(\frac{[BMAL]}{KaP}\right)^{haP}}{1+\left(\frac{[BMAL]}{KaP}\right)^{haP}\cdot\left(1+\left(\frac{[PC]}{KiP}\right)^{hiP}\right)}$
$f_{Per,nutrient}$	$\frac{1+cgc\cdot\left(\frac{[Glucose]}{Kgc}\right)^{hgc}}{1+\left(\frac{[Glucose]}{Kgc}\right)^{hgc}}$
$f_{Rev,clock}$	$\frac{1+g_R\cdot\left(\frac{[BMAL]}{KaR}\right)^{haR}}{1+\left(\frac{[BMAL]}{KaR}\right)^{haR}\cdot\left(1+\left(\frac{[PC]}{KiR}\right)^{hiR}\right)}$
f_{Rev,SCN_cues}	$\frac{1+(1+cneur)\cdot\left(\frac{Neural\_cues}{Kneur}\right)^{hneur}}{1+\left(\frac{Neural\_cues}{Kneur}\right)^{hneur}}$
$f_{Bmal,clock}$	$\frac{1}{1+\left(\frac{[REV]}{KiB}\right)^{hiB}}$
$f_{Cry,clock}$	$\frac{1+g_C\cdot\left(\frac{[BMAL]}{KaC}\right)^{haC}}{1+\left(\frac{[BMAL]}{KaC}\right)^{haC}\cdot\left(1+\left(\frac{[PC]}{KiCp}\right)^{hiCp}\right)}\cdot\frac{1}{1+\left(\frac{[REV]}{KiCr}\right)^{hiCr}}$
$f_{Exo,clock}$	$\frac{\left(\frac{[BMAL]}{KaE}\right)^{haE}}{1+\left(\frac{[BMAL]}{KaE}\right)^{haE}\cdot\left(1+\left(\frac{[PC]}{KiE}\right)^{hiE}\right)}$
$f_{Ins,glucose}$	$\frac{1+g_{gluco}\cdot\left(\frac{[Glucose]}{Kgluc}\right)^{hgluc}}{1+\left(\frac{[Glucose]}{Kgluc}\right)^{hgluc}}$
$f_{Ins,EXO}$	$\frac{1+g_{exo}\cdot\left(\frac{[EXO]}{Kexo}\right)^{hexo}}{1+\left(\frac{[EXO]}{Kexo}\right)^{hexo}}$

Table S2: Transcription functions.

2 Derivation of transcription functions

In order to clarify how we obtain the transcription functions of Table S2, we here show as an example how we derive the transcription function used to describe the dynamics of *Per* mRNA. The transcription of *Per* is controlled by the clock as well as by systemic cues. If the two cues act independently, the transcription function can be written as

$$T_{Per} = f_{Per,clock} \cdot f_{Per,nutrient} \tag{S19}$$

We thus have

$$\frac{dPer}{dt} = vmP \cdot f_{Per,clock} \cdot f_{Per,nutrient} - dmP \cdot Per \tag{S20}$$

The dynamics of *Per* transcription can be described as follows: first, the binding of the activator (here *BMAL1*) on the promotor of the *Per* gene establishes a transcriptionally active state and leads to the synthesis of *Per* mRNA. Then, the repressor complex *PER-CRY* (*PC*) binds to the E-box, which sets the beginning of the repressive state: the transcription of the gene *Per* is then inhibited. This set of reactions can be written as:

$$E0 \overset{k0}{\rightarrow} E0 + Per \tag{S21}$$

$$E0 + BMAL1 \underset{k_{-1}}{\overset{k_1}{\rightleftharpoons}} E1 \tag{S22}$$

$$E1 \overset{k2}{\rightarrow} E1 + Per \tag{S23}$$

$$E1 + PC \underset{k_{-3}}{\overset{k_3}{\rightleftharpoons}} E2 \tag{S24}$$

where *E0* corresponds to the unbound form of the E-box, *E1* to the E-box with BMAL1 bound and *E2* to the E-box with *BMAL1* and *PER-CRY* bound.
The parameters k_1, k_{-1}, k_3 and k_{-3} are binding and unbinding rates while the parameters k_0 and k_2 are transcription rates.
A conservation relation can also be written:

$$E0 + E1 + E2 = cste = Et \tag{S25}$$

If we translate the above reactions into ordinary differential equations by using mass action kinetics, we obtain:

$$\frac{d\,E1}{dt} = k_1 \cdot E0 \cdot BMAL1 - k_{-1} \cdot E1 + k_{-3} \cdot E2 - k_3 \cdot E1 \cdot PC \tag{S26}$$

$$\frac{d\,E2}{dt} = k_3 \cdot E1 \cdot PC - k_{-3} \cdot E2 \tag{S27}$$

$$\frac{d\,E0}{dt} = -k_1 \cdot E0 \cdot BMAL1 + k_{-1} \cdot E1 \tag{S28}$$

Moreover, we have

$$f_{Per,clock} = (k_0 \cdot E0 + k_2 \cdot E1) \tag{S29}$$

By using the conservation relation and the quasi-steady state approximation for Eqs S26, S27 and S28 (i.e. $\frac{d\,E1}{dt}=\frac{d\,E2}{dt}=\frac{d\,E0}{dt}=0$), the equation for the clock-dependent transcription of *Per* becomes

$$f_{Per,clock} = vmP \cdot \left(\frac{\left(1 + g_P \cdot \left(\frac{[BMAL]}{KaP}\right)\right)}{1 + \left(\frac{[BMAL]}{KaP}\right) \cdot \left(1 + \left(\frac{[PC]}{KiP}\right)\right)} \right) \tag{S30}$$

where $g_P = \dfrac{k_2}{k_0}$, $KaP = \dfrac{k_{-1}}{k_1}$, $KiP = \dfrac{k_{-3}}{k_3}$ and $vmP = k_0 \cdot Et$.
Taking into account the cooperative binding to the E-box leads to

$$f_{Per,clock} = vmP \cdot \left(\frac{\left(1 + g_P \cdot \left(\frac{[BMAL]}{KaP}\right)^{haP}\right)}{1 + \left(\frac{[BMAL]}{KaP}\right)^{haP} \cdot \left(1 + \left(\frac{[PC]}{KiP}\right)^{hiP}\right)} \right) \tag{S31}$$

where the cooperativity is characterised by the Hill coefficients haP and hiP.

3 Parameter values

3.1 Parameter estimation

To our knowledge, the kinetic parameters have not been measured in pancreas. Here there are all estimated by parameter estimation methods. We used the Hooke and Jeeves pattern search optimisation method to minimise an objective function defined as the weighted sum of the square of the distance between *in silico* data points and experimental data from mice pancreas [11]. In these experiments, mice were kept in 12h light-dark conditions (DD), fed *ad libitum* and sacrified every 4 hours at ZT0 or ZT12 (with ZT0 corresponding the start of the light period and ZT12 to the start of the dark period). The data we used correspond to clock gene expression and metabolic time profiles. We first normalised the data (that is, divided them by the mean expression of each gene) and then interpolated them by Fourier series to facilitate the adjustment of the model to experimental data. The comparison between *in silico* and experimental time profiles is illustrated in Figure 3 in the main text. In [11], data originate from at least three experiments and are reported as mean +/- SEM. SEM is the standard error of mean which characterises the precision of the estimated mean of the population. Contrary to the standard deviation, it does not represent the variability within the data. To avoid misleading interpretation, SEM is not shown Figure 1 and 3 of the main text.

Parameter values are given in Tables S3-S5. Note that most transcription rates on the one hand and most degradation rates on the other hand lie in the same range except those of *Rev-Erbα* (vmR, dmR). When strongly reducing the value of the synthesis and the degradation rates of *Rev-Erbα* while keeping other parameters at their default value, the robustness of the oscillations during daytime feeding is lost. A possible explanation could be that as the profile of *Rev-Erbα* is far from a sinusoidal one, larger parameters values are needed to describe it. However, we cannot exclude that there are other sets of parameter values for which vmR and vmR are much lower and which would also reproduce the data. We should also point out that given the simplified nature of the model, a single parameter typically encompass various processes (RNA maturation, post-translational modifications, transport).

3.2 Sensitivity to parameter values

One might wonder if the "gene-specific phase shift" property depends on a specific set of parameter values or if it is general for the described model. To answer this question, we investigate how the phase shift of *Bmal1* and *Per* mRNAs is influenced by the variations of a few relevant parameters. Variations in these parameter values modify either the strength of the input of the zeitgebers (light or/and food) or the strength of the different core clock feedback loops. Figure S2 shows that when inverting the feeding schedule:

- the higher the strength of food intake, the more *Bmal1* mRNA shifts (Figure S2A-B).
- the higher the strength of light cues (transmitted through SCN-driven), the less *Bmal1* mRNA shifts (Figure S2C-D).

- the higher the activation of *Per* by BMAL1, the less *Per* and *Bmal1* mRNAs shift . This is probably because in this case, more information about the non-shifting light cues is transmitted to *Per* mRNA through *Bmal1* mRNA (and *Rev-Erb* mRNA) (Figure S2E-F).

- the higher the inhibition threshold of *Rev-Erb* by PER-CRY, the less *Bmal1* shifts. This is probably because in this case, less information about the shifting food cues is transmitted to *Rev-Erb* mRNA and then to *Bmal1* mRNA through *Per* mRNA (Figure S2G-H).

- the higher the inhibition threshold of Bmal1 by REV-ERB, the more *Bmal1* mRNA shifts. This is probably because in this case, less information about non-shifting light cues is transmitted to *Bmal1* mRNA through *Rev-Erb* mRNA (Figure S2I-J).

This analysis thus shows how light and food information are transmitted to the positive arm (here represented by *Bmal1*) and the negative arm (here represented by *Per*) of the core clock. In particular, it indicates that the gene-specific phase shift property does not depend on a specific set of parameter values but can instead easily be obtained by modulating a few key parameters that control how feeding and light cues are transmitted to the different components of the core clock.

	Parameter	Value
Maximal transcription rates [a.u. $\cdot$ h^{-1}]	vmP	0.0194363
	vmR	51.843
	vmB	0.331272
	vmC	0.184884
	vmE	1.3
	$vmE0$	0.5811
mRNA degradation rates [h^{-1}]	dmP	0.1948463
	dmR	59.2343
	dmB	0.137207
	dmC	0.173916
	dmE	0.18
Activation ratio (dimensionless)	g_P	19.8263
	g_R	15.2434
	g_C	4.14061
Activation and inhibition thresholds [a.u.]	KaP	0.00747918
	KaR	0.0572012
	KaC	0.610825
	KaE	0.010016
	KiP	0.419373
	KiR	0.33849
	KiB	0.0527527
	$KiCp$	0.353071
	$KiCr$	8.02667
	KiE	0.49
	Kgc	1.07691
	$Kneur$	0.29268
Hill coefficients (dimensionless)	haP	3.5493
	haR	4.60199
	haC	10.5851
	haE	6.31
	hiP	3.99451
	hiR	9.4383
	hiB	9.44469
	$hiCp$	7.05126
	$hiCr$	1.78207
	hiE	9.69
	hgc	11.4048
	$hneur$	2.16555

Table S3: Parameter values.

	Parameter	Value
Protein degradation rates [h^{-1}]	dpP	0.138646
	dpR	4.8503
	dpB	0.284604
	dpC	1.34809
	dpE	0.42
	$dgPC$	0.0470157
Protein synthesis rates [h^{-1}]	kpP	8.17654
	kpR	1.501
	kpB	0.177396
	kpC	0.172074
	kpE	0.11
Coupling strength (dimensionless)	cgc	2.98
	$cneur$	1.4336

Table S4: Parameter values (cont.).

	Parameter	Value	Units
Association constant	$kass$	0.0206902	[(a.u. · h)$^{-1}$]
Dissociation constant	$kdiss$	0.159696	[h^{-1}]
Constants describing Glucose and Insulin dynamics	kg	3.97	[a.u. · h^{-1}]
	dg	3.97	[h^{-1}]
	di	0.72	[h^{-1}]
	$Kinsu$	0.63	[a.u.]
	$kins$	0.013	[a.u. · h^{-1}]
	$dins$	0.254884	[h^{-1}]
	g_{exo}	10.03	dimensionless
	$Kexo$	0.8	[a.u.]
	$hexo$	4.88	dimensionless
	g_{gluco}	15	dimensionless
	$Kgluc$	1.21	[a.u.]
	$hgluc$	10	dimensionless

Table S5: Parameter values (cont.).

4 Supplementary figures

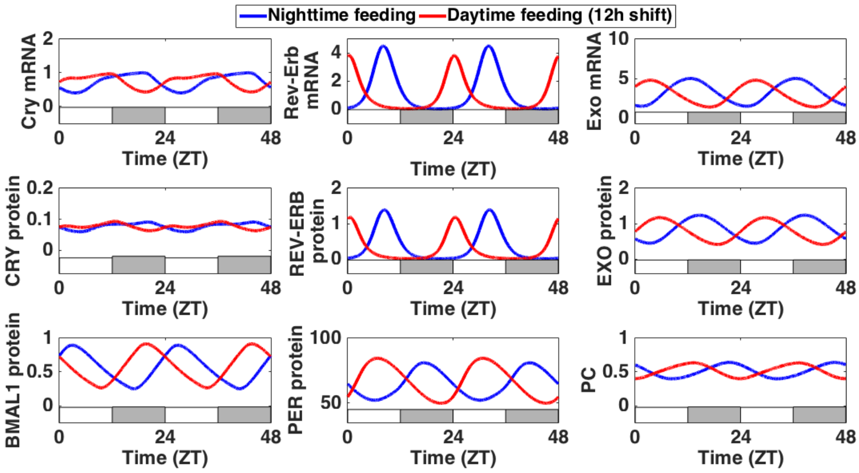

Figure S1: Effect of a shift from nighttime feeding (NF, blue curve) to daytime feeding (DF, red curve) on clock genes mRNA/protein and on EXO mRNA/protein . The alternation of white and grey bars symbolises the LD cycles.

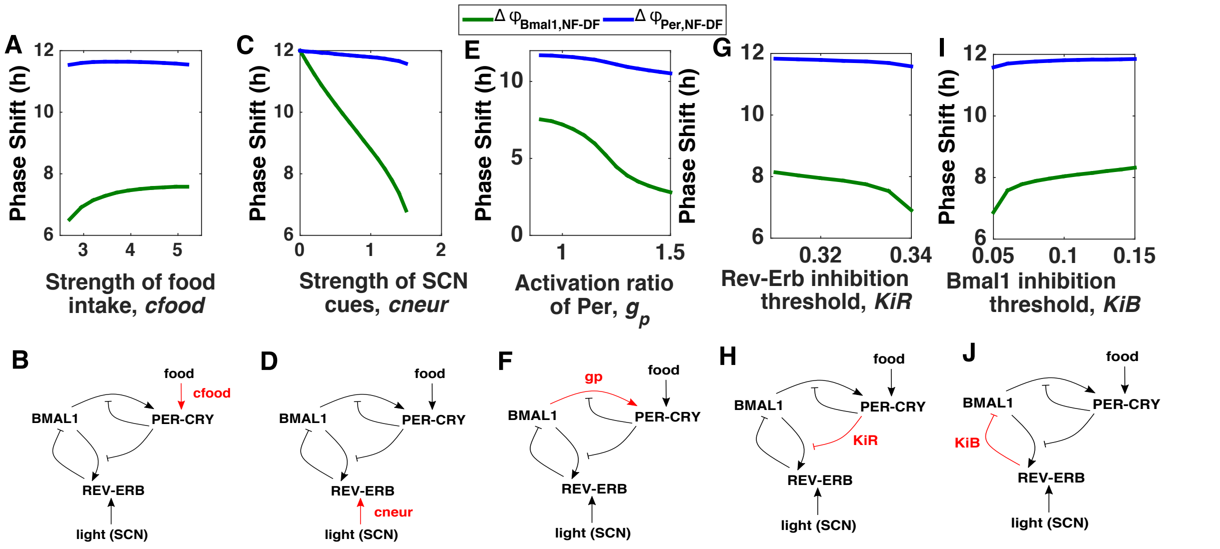

Figure S2: Effect of the variations of a few key parameters on the phase shift of *Bmal1* and *Per* for a 12h shift in food intake.

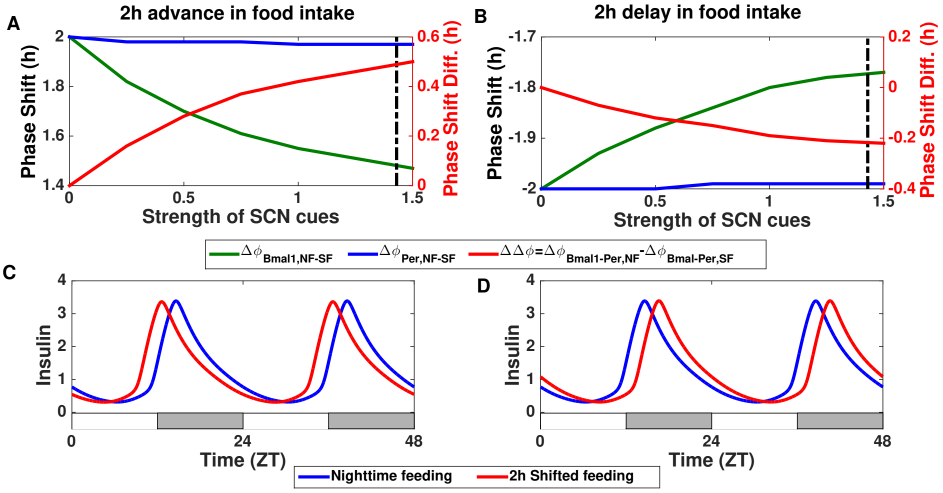

Figure S3: Effect of a 2h phase shift on clock gene expression and insulin secretion. (A) Effect of a 2h advance in food intake as a function of the strength of SCN-driven cues. Left Y-axis: Phase shift as a function of the strength of SCN-driven cues for *Per* mRNA (phase shift in blue) and *Bmal1* mRNA (phase shift in green). The strength of the SCN-driven cues corresponds to the value of the parameter *cneur*. The vertical black dotted line corresponds to the strength of SCN-driven cues used in the rest of the simulations (*cneur*=1.4336). Right Y-axis: The phase shift difference between *Bmal1* and *Per* mRNAs (which corresponds to the phase difference *Bmal-Per* in nighttime feeding (NF) minus the phase difference *Bmal-Per* in the shifted feeding regime) is also represented as a function of the strength of SCN-driven cues (red curve). (B) As in A in the case of a 2h delay in food intake. (C) Insulin levels compared for nighttime feeding (blue curve) and a 2h advance in food intake (red curve). (D) As in C in the case of a 2h delay in food intake. The alternation of white and grey bars symbolises the LD cycles.

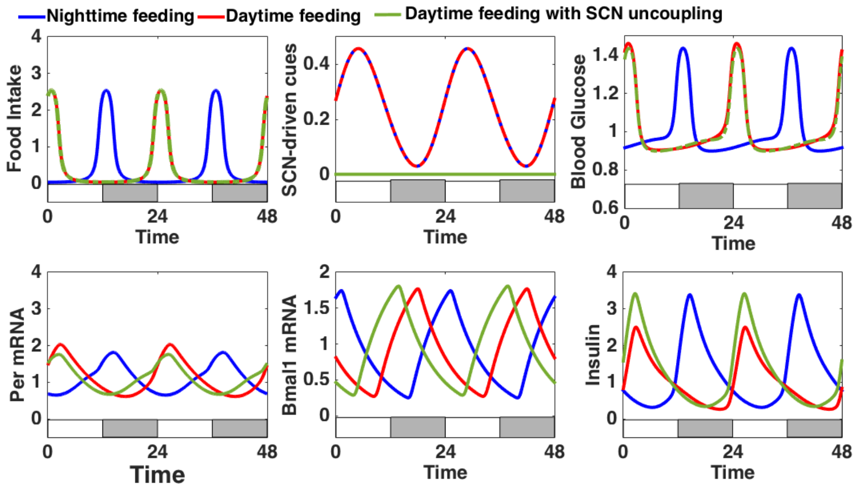

Figure S4: Effect of a shift from nighttime feeding (NF, blue curves) to daytime feeding (DF). Comparison between the assumed physiological situation (red curves) and the case where peripheral clocks would uncouple from the SCN (green curves). The alternation of white and grey bars symbolises the LD cycles.

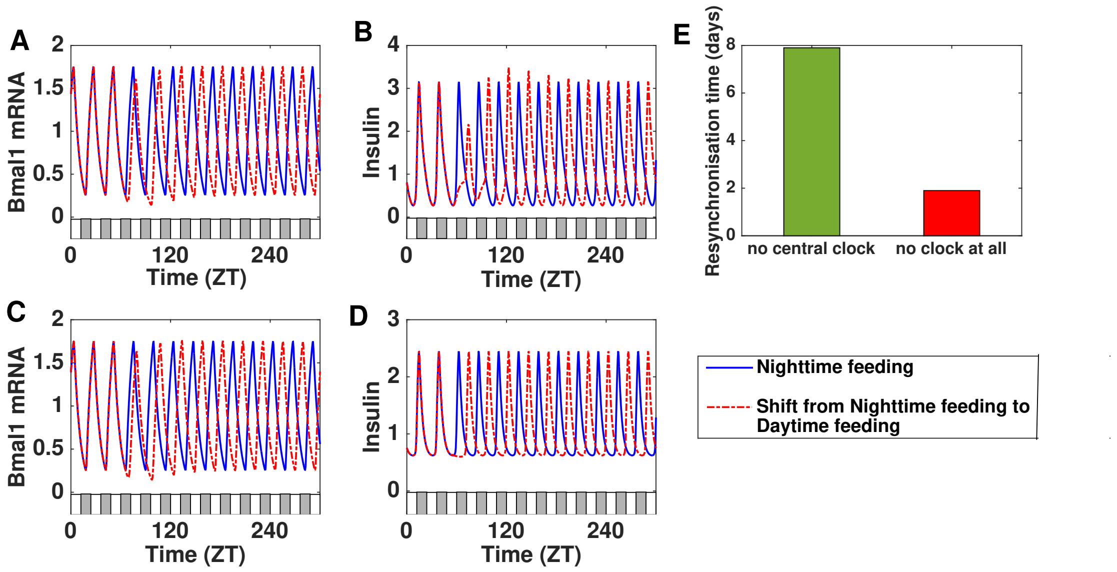

Figure S5: Resynchronisation time to the new feeding schedule (shift from nighttime feeding to daytime after two days) for different architectures, namely "No central clock" condition vs "No clock at all" condition). (A-B) *Bmal1* expression and Insulin levels when the eating schedule is shifted from nighttime feeding to daytime (red curve) in the "No central clock" condition, to be compared with the nighttime feeding (blue curve). (C-D) *Bmal1* expression and Insulin levels when the eating schedule is shifted from nighttime feeding to daytime (red curve) in the "No clock at all" condition, to be compared with the nighttime feeding (blue curve). The alternation of white and grey bars symbolises the LD cycles. (E) Time required to resynchronise to the new feeding schedule (when shifting from nighttime feeding to daytime) compared for the "No central clock" condition (green bar) and the "no clock at all" condition (red bar).

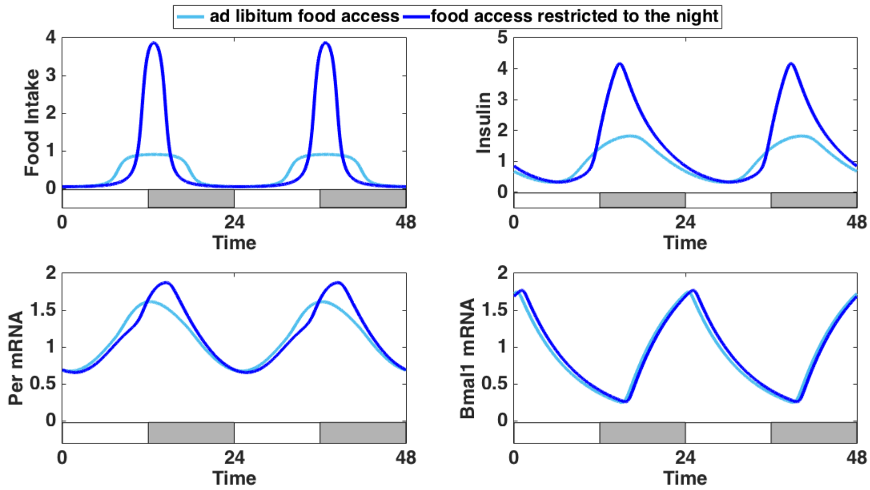

Figure S6: Comparison of clock gene expression and levels of metabolic actors for two different food-access paradigms: *ad libitum* food access (light blue curves) versus food access restricted to the night (dark blue curves). The alternation of white and grey bars symbolises the LD cycles.

References

[1] Bintu, L., Buchler, N. E., Garcia, H. G., Gerland, U., Hwa, T., Kondev, J., and Phillips, R. (2005). Transcriptional regulation by the numbers: models. Current Opinion in Genetics & Development, 15(2):116–124.

[2] Chaix, A., Zarrinpar, A., and Panda, S. (2016). The circadian coordination of cell biology. The Journal of Cell Biology, 215(1):15–25.

[3] Damiola, F., Le Minh, N., Preitner, N., Kornmann, B., Fleury-Olela, F., and Schibler, U. (2000). Restricted feeding uncouples circadian oscillators in peripheral tissues from the central pacemaker in the suprachiasmatic nucleus. Genes & Development, 14(23):2950–2961.

[4] Hatori, M., Vollmers, C., Zarrinpar, A., DiTacchio, L., Bushong, E., Gill, S., Leblanc, M., Chaix, A., Joens, M., Fitzpatrick, J., Ellisman, M., and Panda, S. (2012). Time-Restricted Feeding without Reducing Caloric Intake Prevents Metabolic Diseases in Mice Fed a High-Fat Diet. Cell Metabolism, 15(6):848–860.

[5] Houben, T., Coomans, C. P., and Meijer, J. H. (2014). Regulation of Circadian and Acute Activity Levels by the Murine Suprachiasmatic Nuclei. PLoS ONE, 9(10):e110172.

[6] Ishida, A., Mutoh, T., Ueyama, T., Bando, H., Masubuchi, S., Nakahara, D., Tsujimoto, G., and Okamura, H. (2005). Light activates the adrenal gland: Timing of gene expression and glucocorticoid release. Cell Metabolism, 2(5):297–307.

[7] Koike, N., Yoo, S.-H., Huang, H.-C., Kumar, V., Lee, C., Kim, T.-K., and Takahashi, J. S. (2012). Transcriptional Architecture and Chromatin Landscape of the Core Circadian Clock in Mammals. Science, 338(6105):349–354.

[8] Le Minh, N. (2001). Glucocorticoid hormones inhibit food-induced phase-shifting of peripheral circadian oscillators. The EMBO Journal, 20(24):7128–7136.

[9] Liu, A. C., Tran, H. G., Zhang, E. E., Priest, A. A., Welsh, D. K., and Kay, S. A. (2008). Redundant Function of REV-ERB and and Non-Essential Role for Bmal1 Cycling in Transcriptional Regulation of Intracellular Circadian Rhythms. PLoS Genetics, 4(2):e1000023.

[10] Lowrey, P. L. and Takahashi, J. S. (2004). MAMMALIAN CIRCADIAN BIOLOGY: Elucidating Genome-Wide Levels of Temporal Organization. Annual Review of Genomics and Human Genetics, 5(1):407–441.

[11] Mukherji, A., Kobiita, A., Damara, M., Misra, N., Meziane, H., Champy, M.-F., and Chambon, P. (2015). Shifting eating to the circadian rest phase misaligns the peripheral clocks with the master SCN clock and leads to a metabolic syndrome. Proceedings of the National Academy of Sciences of the United States of America, 112(48):E6691–6698.

[12] Perelis, M., Marcheva, B., Ramsey, K. M., Schipma, M. J., Hutchison, A. L., Taguchi, A., Peek, C. B., Hong, H., Huang, W., Omura, C., Allred, A. L., Bradfield, C. A., Dinner, A. R., Barish, G. D., and Bass, J. (2015). Pancreatic cell enhancers regulate

rhythmic transcription of genes controlling insulin secretion. Science (New York, N.Y.), 350(6261):aac4250.

[13] Qian, J., Block, G. D., Colwell, C. S., and Matveyenko, A. V. (2013). Consequences of Exposure to Light at Night on the Pancreatic Islet Circadian Clock and Function in Rats. Diabetes, 62(10):3469–3478.

[14] Relgio, A., Westermark, P. O., Wallach, T., Schellenberg, K., Kramer, A., and Herzel, H. (2011). Tuning the Mammalian Circadian Clock: Robust Synergy of Two Loops. PLoS Computational Biology, 7(12):e1002309.

[15] Topp, B., Promislow, K., deVries, G., Miura, R. M., and Finegood, D. T. (2000). A model of beta-cell mass, insulin, and glucose kinetics: pathways to diabetes. Journal of Theoretical Biology, 206(4):605–619.

[16] Travnickova-Bendova, Z., Cermakian, N., Reppert, S. M., and Sassone-Corsi, P. (2002). Bimodal regulation of mPeriod promoters by CREB-dependent signaling and CLOCK/BMAL1 activity. Proceedings of the National Academy of Sciences, 99(11):7728–7733.

[17] Vieira, E., Marroqu, L., Batista, T. M., Caballero-Garrido, E., Carneiro, E. M., Boschero, A. C., Nadal, A., and Quesada, I. (2012). The Clock Gene *Rev-erb* Regulates Pancreatic -Cell Function: Modulation by Leptin and High-Fat Diet. Endocrinology, 153(2):592–601.

[18] Woller, A., Duez, H., Staels, B., and Lefranc, M. (2016). A Mathematical Model of the Liver Circadian Clock Linking Feeding and Fasting Cycles to Clock Function. Cell Reports, 17(4):1087–1097.

[19] Yang, X., Downes, M., Yu, R. T., Bookout, A. L., He, W., Straume, M., Mangelsdorf, D. J., and Evans, R. M. (2006). Nuclear Receptor Expression Links the Circadian Clock to Metabolism. Cell, 126(4):801–810.
